# Needs Analysis for a Parenting App to Prevent Unintentional Injury in Newborn Babies and Toddlers: Focus Group and Survey Study Among Chinese Caregivers

**DOI:** 10.2196/11957

**Published:** 2019-04-30

**Authors:** Peishan Ning, Deyue Gao, Peixia Cheng, David C Schwebel, Xiang Wei, Liheng Tan, Wangxin Xiao, Jieyi He, Yanhong Fu, Bo Chen, Yang Yang, Jing Deng, Yue Wu, Renhe Yu, Shukun Li, Guoqing Hu

**Affiliations:** 1 Department of Epidemiology and Health Statistics Xiangya School of Public Health Central South University Changsha China; 2 Department of Psychology University of Alabama at Birmingham Birmingham, AL United States; 3 Department of Biostatistics College of Public Health and Health Professions University of Florida Gainesville, FL United States; 4 Department of Occupational and Environmental Health Xiangya School of Public Health Central South University Changsha China; 5 Information and Network Center Central South University Changsha China

**Keywords:** injury, child, mobile health, education, intervention, parenting, mhealth

## Abstract

**Background:**

With the growing popularity of mobile health technology, app-based interventions delivered by smartphone have become an increasingly important strategy toward injury prevention.

**Objective:**

This study aimed to develop a framework supporting the design of an app-based intervention to prevent unintentional injury, targeted for caregivers of Chinese children aged 0 to 6 years.

**Methods:**

A theory-based mixed-method study, including focus groups and Web-based quantitative survey, was performed. Adult caregivers who care for children aged 0 to 6 years and own a smartphone were recruited into 2 sequential stages of research. First, focus groups were conducted among the caregivers at community health care centers and preschools from December 2015 to March 2016. Focus groups (8-10 participants per group) explored awareness, experiences, and opinions of caregivers toward using an app to prevent unintentional injury among children. Second, based on the focus groups findings, a Web-based quantitative survey was designed and distributed to caregivers in November 2016; it collected information on specific needs for the app-based intervention. Thematic analysis and quantitative descriptive analyses were performed.

**Results:**

In total, 12 focus groups were completed, involving 108 caregivers. Most participants expressed a strong desire to learn knowledge and skills about unintentional child injury prevention and held positive attitudes toward app-based interventions. Participants expressed multiple preferences concerning the app-based intervention, including their contents, functions, interactive styles, installation and registration logistics, and privacy protection and information security. Following the focus groups, 1505 caregivers completed a WeChat-based quantitative survey, which generated roughly similar results to those of focus groups and added numerical metrics concerning participants’ preferences on what to learn, when to learn it, and how to learn it. A detailed framework was established involving 5 components: (1) content design, (2) functional design, (3) interactive style, (4) installation and registration logistics, and (5) privacy protection and information security, and 15 specific requirements.

**Conclusions:**

We developed a framework that can be used as a guide to design app-based interventions for parents and caregivers, specifically for unintentional injury prevention of children aged 0 to 6 years.

## Introduction

### Background

Unintentional injury is a serious public health problem among children aged 0 to 6 years in China. In 2017, over 20,000 Chinese children younger than 5 years died of unintentional injury, and nearly 2.4 million children required emergency or outpatient care because of unintentional injury [1].

Parenting interventions can substantially reduce child unintentional injury risk by improving caregivers’ safety knowledge and perceptions about the risk for injury to their children and through adoption of safety equipment and practices [2-5]. For a variety of reasons, implementation of such interventions has been inadequate in low- and middle-income countries such as China [6,7]. In fact, 2 domestic studies from China indicate that less than 20% of caregivers had ever received education concerning unintentional injury prevention for children aged 0 to 6 years or attended a professional prevention course on the topic [8,9].

Encouragingly, the mobile health (mHealth) movement to deliver health interventions through technology has potential to overcome this barrier to parent education for child injury prevention. The number of smartphone users in China has grown quickly over the past decade, with recent estimates suggesting that about 403 million adults aged 20 to 39 years (95% of the Chinese population in that age group) accessed the internet through mobile phones in December 2017 [10,11]. Compared with traditional health education methods delivered in person by professionals, mHealth interventions are low-cost and easy-to-implement, allow interactions between users and providers, and can be accessed anonymously with flexibility at any time and any place [12-14].

Recently, app-based interventions have been developed to prevent or reduce unintentional injury risk in specific injury domains, including sports injuries [15], fire injuries [16], road traffic injuries [16,17], falls [18], and burns [19]. Some app-based interventions have been critiqued, however, for failing to meet basic principles for effective prevention programs such as being based in theory or being tailored to risks in the target population [20].

Multiple health behavior change theories can be used in the development of an app to reduce injuries. The Theory of Planned Behavior (TPB) offers a social psychological theory that fits nicely, as it is used to interpret and predict why individuals perform specific behaviors [21]. A recent systematic review concluded that TPB is an effective framework to identify and understand child and adolescent nutrition–related behaviors. It found, for example, that attitude was strongly related to dietary behavioral intention (mean *r*=0.52) and intention was the most common predictor of behavior performance (mean *r*=0.38) [22]. Interventions grounded in TPB must attend to topics such as the individual’s attitudes toward using an app-based intervention and changing their behavior as a result of that app, the individual’s subjective norms about behavior that should be conducted, and the individual’s perception of how much they can control their own behavior surrounding the health area of interest.

Computer science theories are also critical to the development of an app-based intervention. The Framework for the Rational Analysis of Mobile Education (FRAME) model offers a theoretical grounding in computer science to guide the development of mHealth learning programs. The FRAME model considers 3 aspects of mobile learning: usability of the device and app, capacities of the learners, and social interaction between users. Regarded as a comprehensive model to develop and implement mobile learning, attention to the components of the FRAME model allows app development to proceed with attention to all relevant components of user learning [23].

### Objectives

We, therefore, conducted a mixed-method study to establish a theory- and need-based framework that would support and lead to the design of an app-based intervention for child injury prevention to be used by Chinese caregivers of children aged 0 to 6 years. All assessment protocols were grounded in TPB and the FRAME model and were designed to gather information that would be valuable for the design of app-based health interventions focused on improving parenting among Chinese caregivers to reduce child injury risk.

## Methods

### Study Design

Grounded in TPB and the FRAME model [21,23], we designed a 2-step sequential mixed-method study. The TPB suggests that an effective app parenting intervention must offer strategies to alter the attitudes, subjective norms, and perceived control to engage in actions that will improve the safety of children. We targeted several aspects of these strategies in our inquiries to caregivers concerning their preferences in the design of an app-based intervention. The FRAME model addresses users’ preferences for app usability, the capacity of the app users, and the social interactions between app users; we addressed these aspects of app functioning also in the inquiries to caregivers.

To gather qualitative data first, focus groups were conducted to explore the experiences and preferences of adult caregivers on using an app-based intervention to prevent unintentional injury among children younger than 7 years. Next, focus group findings were used to guide development and implementation of a Web-based survey to quantify key needs for the app-based interventions (eg, frequency and length of content, variety and types of forms of learning, and duration of app-based learning).

### Focus Group

#### Participants

Participants were recruited for focus groups using purposive sampling. Eligible participants included primary caregivers of 1 or more children younger than 7 years and who used a smartphone regularly. No exclusion criteria applied beyond the age of children that caregivers looked after.

Caregivers of children aged 0 to 3 years were recruited primarily from community health centers. Caregivers of children aged 4 to 6 years were recruited from preschools. To maximize sampling variation, we sampled from a range of preschool types (including both public and private) and geographic locations (including varied socioeconomic status and living areas). Focus group members represented both sexes and a range of household incomes, ages, and levels of education.

#### Setting

Focus groups were completed between December 2015 and March 2016 in Changsha, China. A semistructured discussion guide was developed and refined through pilot testing first among the research group and then with an independent focus group of caregivers of children aged 0 to 3 years (Multimedia Appendix 1). Informed consent was obtained from all participants before each focus group began.

Broadly, discussions were organized to concentrate on perceptions and opinions about unintentional child injury prevention and app-based injury interventions as well as on issues of designing app-based intervention (eg, content, function, interfaces, and data security). To support discussion about intervention interfaces, the facilitators prepared a series of slides that illustrated different styles of interface designs.

When each focus group discussion concluded, participants completed short paper-based questionnaires that collected information on sociodemographic characteristics for both caregivers and their children and unintentional injury history for their children.

Following scholarly recommendations to organize focus groups [24,25], each focus group consisted of 8 to 10 participants and lasted for 60 to 90 min. A total of 3 trained facilitators led the discussion of all groups; 1 served as moderator and the other 2 took extensive discussion notes and supported the moderator as needed to organize the discussion. All focus group discussions were audio-taped, transcribed, and then reviewed before the next focus group. This allowed facilitators to include new concepts or opinions and exclude old but less relevant subtopics iteratively. Focus groups were concluded when no new concepts and opinions emerged.

#### Data Analysis

Focus group audiotapes were transcribed verbatim and checked for accuracy. All participant names were removed to permit anonymized analysis. The transcripts were then analyzed using thematic analysis strategies [26]. First, 2 researchers spent several days familiarizing themselves with all transcripts and generating initial codes independently. Second, the 2 coders combined initial codes and organized them into themes based on the discussion guideline, concordance between codes, and the underlying theory. Third, the 2 researchers’ proposed themes were combined and re-evaluated to ensure no major themes from the discussions were omitted. Finally, group discussions of the research group (including 2 coders) were held to achieve consensus on any themes where the 2 researchers held differing views. Transcript analysis and coding were completed using MAXQDA 12.0 (qualitative data analysis software by VERBI GmbH).

### Web-Based Survey

#### Participants

Study participants for the Web-based survey component of the study were recruited through snowball sampling. Inclusion criterion matched those of the focus groups: caregivers of children younger than 7 years who owned a smartphone. No exclusion criteria applied beyond the age of children that caregivers looked after.

To maximize reach to the target population, we used WeChat, the most popular social media communication platform in China, with an average of 889 million active users each month [27], to conduct the survey. Specifically, the research team sent a study recruitment message through an official WeChat account known as La Ma Xue Yuan (School for Young Mothers), which includes 9290 users throughout China (mostly caregivers of young children). This message included an invitation letter and the questionnaire. Participants consented to participate online and then completed and submitted the questionnaire. Participants were also encouraged to share the message with their social network, many of whom met the inclusion criterion, allowing us to “snowball” to a larger sample size. The Web-based survey remained open until the number of completed questionnaires plateaued.

#### Setting

The Web-based survey was completed in November 2016. It was developed based on the results of the focus groups, grounded in TPB and especially the FRAME model, and finalized through pilot testing with 20 caregivers. The survey included items assessing demographic characteristics, history of child unintentional injury, prior learning experiences about child unintentional injury prevention, and preferred learning contents (Multimedia Appendix 2). To avoid repeated questionnaire completion, we restricted the survey to a single response from each smartphone. To encourage participation in the Web-based survey portion of the study, raffle prizes with cash incentives were distributed at a probability of 1/3 after participants completed the survey.

#### Data Analysis

The collected questionnaires were screened to exclude those completed by caregivers who did not meet the inclusion criteria. Proportions were then calculated to describe preferred contents of the app-based intervention, forms of app-based learning, preferred learning time, and frequency and duration for using app-based learning. Chi-square test examined group differences across groups. Data analysis was performed using the Statistical Product and Service Solutions (SPSS 18.0; IBM Corporation). *P*<.05 was considered to be statistically significant.

### Ethical Considerations

The protocol was approved by the ethics committee of the Institute of Clinical Pharmacology of Central South University. This study was conducted, analyzed, and reported according to the consolidated criteria for reporting qualitative research (COREQ) [28] and the checklist for reporting results of internet E-survey (CHERRIES [29]; Multimedia Appendix 3). All participants were informed about the study and provided informed consent before participating in the research. All data were analyzed anonymously.

## Results

### Focus Group Results

#### Participants

In total, 12 focus groups were organized; together they included 108 caregivers (90 parents, 6 grandparents, and 12 preschool teachers; Table 1). The largest portions of participants were female (96/108, 88.9%) and aged 20 to 39 years (94/108, 87.0%). Among the parent and grandparent participants, the caregivers supervised 96 children who ranged in age from 0 to 6 years, with the largest portions of children aged 4 to 6 years (35/96, 37%) and 0 to 1 year (34/96, 35%). The ratio of boys to girls supervised was 1.04 (Table 1).

#### Attitude and Behavioral Intention Toward Child Unintentional Injury Prevention

Most caregivers reported a strong desire to learn unintentional injury prevention strategies for children aged 0 to 6 years. Many participants mentioned that they felt unintentional injuries were largely preventable and that they had a strong intention to learn knowledge and skills that would help them prevent unintentional injuries to children under their care. A typical opinion was as follows:

We definitely want to learn it[knowledge about unintentional injury prevention for children ages 0-6 years]. From my point of view, it would be very useful and valuable for us [to learn that information].

Participant #DWT-A-01

**Table 1:** Demographic characteristics of caregiver participants.

Variable			Focus group participants, n (%)	Web-based survey participants, n (%)
**Caregivers**				
	Total		108 (100.0)	1505 (100.00)
	**Sex**			
		Male	12 (11.1)	687 (45.65)
		Female	96 (88.9)	818 (54.35)
	**Age group (years)**				
		≤19	2 (1.9)	33 (2.19)
		20-29	48 (44.4)	805 (53.49)
		30-39	46 (42.6)	604 (40.13)
		40-49	8 (7.4)	62 (4.12)
		≥50	4 (3.7)	1 (0.07)
	**Relationship with children**			
		Parent	90 (83.3)	—^a^
		Grandparent	6 (5.6)	—^a^
		Preschool teacher	12 (11.1)	—^a^
**Children^b^**				
	**Sex**			
		Male	49 (51)	859 (57.08)
		Female	47 (49)	646 (42.92)
	**Age group (years)**				
		≤1	34 (35)	205 (13.62)
		2-3	27 (28)	602 (40.00)
		4-6	35 (37)	698 (46.38)

^a^Information was not collected.

^b^Children of the participant supervisors (90 parents and 6 grandparents) in the focus groups, excluding participants who were preschool teachers.

Beyond wanting to learn knowledge about child injury prevention, the majority of focus group participants expressed the opinion that they could use a smartphone app to learn that knowledge. The participants believed that an app developed by a professional team to address child injury prevention would be credible and could be created to include sufficient content for their learning. They also expressed the opinion that an app may be a more convenient educational method than alternatives, such as written brochures or social networks like WeChat, to search for injury prevention content when they wanted it:

Compared to other options, an app-based intervention developed by a professional team would provide rich content. Thus, it will be convenient to search for the knowledge we need. For example, knowledge we can obtain in WeChat is hard to retrieve when we need it again.

Participant #YL-B-06

A few participants expressed some concerns about the utility of a child injury prevention app. They worried about the possible difficulty in the installation and registration process and the possibility that it would use too much memory space on their smartphones, but they said they would accept the app if these problems were addressed.

#### Content of App-Based Intervention

Participants mentioned with some frequency 10 major causes of child unintentional injury that would be worth including in the app: exposure to animate mechanical forces (including animal bites and being trampled or bumped by other people); exposure to inanimate mechanical forces (including being pinched between 2 surfaces, such as in the doors of elevators, or cut or punctured by sharp objects); falls; contact with heat and hot substances; exposure to smoke, fire, and flames; transport crashes; unintentional threats to breathing that create suffocation risk; unintentional poisoning by and exposure to noxious substances; unintentional drowning and submersion; and exposure to electrical currents. Example statements appear below:

I really worry about injury from falls because children always like jumping from high places to lower places, which highly increases the risk of getting injured. In addition, road crashes are another important injury that I want to learn about, because many children run across or play in the street even when they see a car nearby.

Participant #DWT-B-03

Falls and burns are common types of unintentional injury for children ages 0-6 years at home. Relatively speaking, their harm to children is not as great as other types, such as electricalcurrents, which is one of the most dangerous injury causes for children.

Participant #HHY-B-04

Participants also recommended 3 facets of content design that could potentially increase the use of app-based intervention by caregivers: (1) providing professional and believable content to gain the trust of app users, (2) using plain language to make the contents easy to learn, and (3) providing easy-to-implement interventions to improve the practicability of applying lessons from the app-based intervention.

#### Forms of Learning Delivered by an App-Based Intervention

Several forms of learning from an app were proposed by the participants. Most frequently mentioned were short written alarms or warnings with pictures, cartoon vignettes, video testimonials, and interactive games. The participants believed these 4 forms would make learning easier and more engaging. Participants also mentioned the desire to learn the knowledge interactively with their children, a strategy they felt might maximize the effectiveness of the injury prevention program:

Pictures and video are easy to understand for both adults and children and might be the best way to disseminate the prevention knowledge. I also think both caregivers and children need to learn unintentional injury prevention. The easier the learning form, the better the learning outcome. In addition, it is better to include short warning words in the pictures to more effectively stop the children from adopting dangerous behaviors.

Participant #DWT-B-01

#### Timing for App-Based Learning

There was some variation across caregivers, but the general consensus was that they preferred the opportunity to use the app twice a week, for between 2 to 5 min at each session. As an example, a participant made the following statement:

The duration of the app-based learning should not be too long. I think “no more than 5 minutes” is fine for me because over five minutes learning would make me dizzy.

Participant #MWD-B-10

Participants also recommended evening as the best time of the day to interact with the app:

For me, it is particularly suitable to learn injury prevention knowledge at 8 or 9 o'clock in the evening. At that time, I have finished housework and my child hasfallen asleep.

Participant #MWD-B-09

Some participants felt the length of time they used the app would depend greatly on how much they enjoyed using it and that tangible cases and visual attraction would improve the authority and authenticity of the training, increasing their desire to use the app.

#### Functional Design of the App-Based Intervention

Almost all focus group participants mentioned interactive features as an indispensable function of the app-based intervention. They explained that interactive portions would maintain the attention of app users and increase the effectiveness of the intervention. Web-based chats, forums, and message boards were suggested as ways to implement interactive processes:

Web-based chatting is the best way to solve the problems that we encounter in our lives.

Participant #HHY-A-01

As we know, our kids may encounter various unintentional injuries in daily life. If the app intervention can set up a module listing possible ways to prevent common injury causes, it would be great. In addition, it would be attractive to me if the app had a forum in which I could discuss these topics with other caregivers who confront the same questions.

Participant #YL-C-06

In addition, a customer service agent who was accessible through Web-based chatting and a frequently asked questions module was suggested by the caregivers participating in the focus groups.

Beyond interactive portions of the app, most caregivers in the focus groups felt that a survey with feedback would be an important function of the app-based intervention. Such a component would allow app users to be aware of children’s unintentional injury risk in their homes and to obtain tailored professional recommendations to prevent injuries to their children. Both Web-based and printed questionnaire surveys were recommended as appropriate approaches to obtain feedback from professionals.

In addition, some participants suggested surveys and feedback should be scheduled to be brief (eg, 5 min to complete each survey) and to be repeated regularly (eg, 3-4 times per year). Parents felt their responses might change as children develop new skills with older age, and therefore, tailored information that coincides with their children’s development would be valuable. Participants recommended that questionnaires be based on items with categorical response options (eg, true or false) that could be responded to quickly. A few caregivers suggested providing survey participants with small gifts or bonuses to maintain their adherence to the intervention. Furthermore, to increase compliance in completing Web-based surveys, participants suggested the use of short message service text message reminders:

I’d like to use Web-based questionnaires to send feedback. I think survey questions with single options are much better than open-ended survey questions because I really do not know what should be filled in in many cases.

Participant #HHY-B-03

Both Web-based and printed questionnaire surveys are acceptable for me, but a Web-based questionnaire survey is preferred since it is more convenient and takes less time than a printed questionnaire survey. I probably would agree to complete a paper questionnaire survey if I received small gifts after I completed it.

Participant #HHY-B-04

Some caregivers extended the survey idea further, suggesting that it would be valuable to have the app customized to their preferences and priorities in terms of content, form, frequency, and interface of the app. As an example, preventive content concerning riding a bicycle may not be of interest or relevant to caregivers whose children are younger than 3 years, as most Chinese children do not learn to ride bicycles until they are at least 4 years old. Thus, parents suggested the app be tailored so that such segments would be omitted in their version of the program.

#### Design of the Interface

Participants stated that they would like to choose the app interface (eg, color and style) based on their preferences. During the group discussion, the moderator demonstrated various interfaces, including the layout of 3 existing apps, to obtain participants’ opinions on the app design. Of the 3 options offered by the research team, most caregivers chose a cartoon style. A few caregivers preferred a simple interface with 3 replaceable pages, and fewer still selected a simple interface with only 1 page.

#### Installation and Registration

Caregivers strongly suggested the app should take up only a small amount of memory space on their smartphone and that it should require a simple registration procedure. They believed a user manual or help module might assist them with app use, and they requested an informational module that described the purpose, details, and benefits of the project before starting to use the app-based intervention.

#### Privacy and Data Security

Many caregivers expressed privacy and security concerns about using the app, especially if they were requested to provide sensitive information about their children (eg, home address and activities and locations where children engage in those activities). They did state that they would trust apps downloaded from officially certified app shops or promoted by official agencies such as preschools.

### Web-Based Survey Results

#### Participants

In total, 1505 valid questionnaires were collected through the Web-based survey, including 687 from men (45.65%) and 818 from women (54.35%). The respondents supervised 807 children aged 0 to 3 years (53.62%) and 698 children aged 4 to 6 years (45.97%). The proportion of male to female children supervised was 1.33 (Table 1).

#### Content of App-Based Intervention

Of the 1505 participants who completed the Web-based survey, 1313 (87.24%) expected the app would teach them relevant knowledge concerning unintentional injury prevention for children aged 0 to 6 years. Participants felt it would be valuable to learn knowledge about preventing the 10 major causes of child unintentional injury at different rates: contact with heat and hot substances (534/1313, 40.67% felt it would be valuable to learn); inanimate mechanical forces (including being pinched between 2 surfaces, such as in the doors of elevators, or cut or punctured by sharp objects; 520/1313, 39.60%); falls (449/1313, 34.20%); transport crashes (362/1313, 27.57%); unintentional threats to breathing (361/1313, 27.49%); exposure to animate mechanical forces (317/1313, 24.14%);exposure to smoke, fire, and flames (210/1313, 15.99%); unintentional poisoning by and exposure to noxious substances (146/1313, 11.12%); unintentional drowning and submersion (113/1313, 8.61%); and exposure to electrical currents (108/1313, 8.23%). The differences were statistically significant (χ^2^_9_=989.7; *P*<.05; Table 2).

**Table 2:** Number of participants who expressed a desire to learn knowledge about preventing major types of child unintentional injury causes.

Cause of injury	n (%)
Contact with heat and hot substances	534 (40.67)
Exposure to inanimate mechanical forces	520 (39.60)
Falls	449 (34.20)
Transport crashes	362 (27.57)
Unintentional threats to breathing	361 (27.49)
Exposure to animate mechanical forces	317 (24.14)
Exposure to smoke, fire, and flames	210 (15.99)
Unintentional poisoning by and exposure to noxious substances	146 (11.12)
Unintentional drowning and submersion	113 (8.61)
Exposure to electrical currents	108 (8.23)

#### Forms of App-Based Learning

The 5 most frequent learning forms that participants stated they would like to use were as follows: (1) short written alarms or warnings with pictures, (2) video testimonials, (3) cartoon vignettes, (4) pictures, and (5) interactive games, which were, respectively, preferred by 62.30% (818/1313), 54.53% (716/1313), 40.67% (534/1313), 38.31% (503/1313), and 28.03% (368/1313) of respondents (note that respondents were permitted to select multiple preferred learning forms). Differences were significant (*χ*^2^_4_=393.0; *P*<.05).

#### Preferred Times, Frequency, and Duration for Using App-Based Learning

Caregivers varied in their preferred frequency for using an app to learn about unintentional child injury prevention. Just over half the sample (705/1313, 53.69%) preferred to use the app twice a week, followed by once a week (337/1313, 25.67%) and once a day (215/1313, 16.37%). Differences were significant (*χ*^2^_2_=455.4; *P*<.05). Participants were approximately evenly split concerning their preference to use the app in the evening (376/1313, 28.64%), afternoon (332/1313, 25.29%), or noontime (265/1313, 20.18%; *χ*^2^_2_=25.2; *P*<.05). Preferred durations for each session of app-based learning were 6 to 10 min (488/1313, 37.17%), greater than or equal to 11 min (418/1313, 31.84%), and 3 to 5 min (334/1313, 25.44%; *χ*^2^_2_=42.0; *P*<.05).

### Suggested Framework to Design the App Based on Focus Group and Web-Based Survey

On the basis of responses from the focus groups and the Web-based survey, as well as the 3 principles of TPB (attitudes toward the behavior, subjective norms, and perceived behavioral control) and the 3 aspects of the FRAME model (learner, devices, and social aspects), we developed a framework to guide design of an app to teach caregivers knowledge about preventing child unintentional injury. The design included 5 primary components: (1) content design, (2) functional design, (3) interface design, (4) installation and registration, and (5) privacy and data security, plus 15 subcomponents. Details, including the theoretical basis for each recommendation of the participants, appear in Table 3.

**Table 3:** Framework for developing an app to teach knowledge about preventing child unintentional injury to Chinese caregivers of children aged 0 to 6 years.

Requirements		Description	Theoretical basis
**Content, form, time, frequency, and duration of knowledge dissemination**				
	Content	Ten major unintentional injury causes: contact with heat and hot substances; inanimate mechanical forces; falls; transport crashes; unintentional threats to breathing; animate mechanical forces; exposure to smoke, fire, and flames; unintentional poisoning by and exposure to noxious substances; unintentional drowning and submersion; and exposure to electrical currents, with the suggested proportions for knowledge disseminations of these 10 injury causes at the suggested proportions of 5:5:4:4:4:3:2:1:1:1	Learner aspect (FRAME^a^ model)
		Tied to theory-based goals to improve attitudes, alter subjective norms, and increased perceived control to perform child unintentional injury prevention behaviors	Attitudes, subjective norms, and perceived control (TPB^b^)
	Form	Short written alarms or warnings with pictures, cartoon vignettes, video testimonials, and interactive games, with the suggested proportions of 2:2:1:1 across the 4 forms	Learner aspects (FRAME model)
	Time	Preferred time to learn (evening, afternoon, or noon)	Learner aspects (FRAME model)
	Frequency	Twice a week	Learner aspects (FRAME model)
	Duration	No more than 5 min per time	Learner aspects (FRAME model)
	Other attributes	Professionally disseminated contents	Learner aspects (FRAME model)
		Using plain language	Learner aspects (FRAME model)
		Easy to implement	Learner aspects (FRAME model)
**Functions**			
	Interactive style	Regular communication between users and experts through online chat, forums, and message boards; frequently asked questions module; and Web-based customer service agents and ask-and-answer service	Social aspects (FRAME model); attitude and subjective norms (TPB)
	Survey and feedback	Use Web-based and printed questionnaires and short message service text message reminders for surveys and to collect feedback, no more than once every 2 months	—^c^
		After each survey, motivate users through virtual rewards	—
	Personalized customization	Allow personalized customization of contents, forms, frequency, and interface of app intervention	Learner aspect (FRAME model)
**Design of the interface**			
	Interface design	Offer several choices of app interfaces: cartoon interface preferred for default	Device aspect (FRAME model)
**Installation and registration**			
	Registration	Simple app registration procedure	Device aspect (FRAME model)
	Memory space	Minimize the size of app so smartphone storage is not used excessively	Device aspect (FRAME model)
	Informed background	Provide informational background to users’ informed consent process, so they understand before downloading and using the app intervention	Device aspect (FRAME model)
	Manual and help module	Provide a user manual and help module	Device aspect (FRAME model)
**Privacy protection and data security**			
	Personal privacy and data security	Do not collect sensitive individual or family information such as name, home address, or family income	Device aspect (FRAME model)
		Ensure safe sharing and storage of data, for example, by using an individualized password	Device aspect (FRAME model)

^a^FRAME: Framework for the Rational Analysis of Mobile Education.

^b^TPB: Theory of Planned Behavior.

^c^Not applicable.

## Discussion

### Principal Findings

Using a mixed-method study, we explored the preferences of caregivers of Chinese children aged 0 to 6 years for an app offering knowledge about child unintentional injury prevention. Study results helped us establish a framework for the app that includes 5 primary components (content design, functional design, interface design, installation and registration, and privacy and security) and 15 subcomponents focused on educating Chinese caregivers on child unintentional injury prevention strategies. The framework in Table 3 lists details to design the app, including the theoretical basis for those details.

### Comparison With Prior Work

In addition to replicating previous work underlying the urgency to implement unintentional injury prevention education strategies for caregivers of young children in China [9], this study explored caregivers’ attitudes and preferences for app-based learning, offering comprehensive and valuable guidance for the design of an app-based intervention. A few previous studies, generally based on small-sample qualitative designs, proposed some fragmented recommendations on the design of health-related apps that parallel some of our findings [30-34]. For example, Gkatzidou et al [30] suggested that privacy and security, credibility, user journey support, and the task-technology-context fit from the patient’s perspective should be considered in the design of health care apps to maximize their acceptability. Similarly, Curtis et al [34] reported preferences for a healthy eating app targeting parents about child weight management; they identified 4 main themes for app design: app features, time saving and convenience, aesthetics, and gamification.

Our findings agree to a large extent with previous work in the design of app interventions, as we uncovered user preferences to emphasize a user-centered design [35,36], including particular emphasis on the importance of inclusion of games [35] and on the protection of user privacy [36].

### Implications

This study has important implications. First, the research will lead to the development of an app designed to help caregivers learn how to reduce injury risk among their children. Although mobile device interventions are most effective when they are based upon theory [37], many existing health-related mobile apps do not incorporate features derived from evidence-based theoretical frameworks, health behavior change theories, and clinical guidelines [38,39]. We propose research grounded in theory that will lead to the development of an evidence-based intervention program. More broadly, the framework we present may prove valuable to guide the design of app-based interventions to target other diseases and injuries in China, as users’ preferences are likely to be similar across content areas. With cultural tailoring, it might also be useful in other countries, and ultimately, the recommendations could facilitate using smartphone technology to improve public health in China and beyond. Furthermore, our methodological strategies proved useful and could be replicated. The 2-step mixed-method approach could be extended to guide the development of theory-driven frameworks or interventions for various health issues in various cultural contexts.

### Strengths

Our study has 2 strengths. First, we adopted a mixed-method design with large sample sizes. We conducted a rigorous mixed-method study and analyzed data and reported results in accordance with COREQ [28] and CHERRIES [29] guidelines. In particular, we adopted the principles of an effective intervention program (eg, implementing various learning methods, sufficient dosage, theory driven, and appropriately timed) [20] and used the TPB [21] and the FRAME model [23] to design the semistructured discussion guide for focus groups. Use of the TPB and the FRAME model ensured that the framework we proposed both addresses the needs of users (FRAME) and creates a situation that will optimally encourage appropriate behavior change among Chinese caregivers and yield reduced risk of unintentional injury among their young children (TPB).

Second, we implemented rigorous science to develop the focus group guides and the questionnaire for the Web-based survey. Methodological strategies included 2-round discussions within the research member group and pilot tests to improve the experimental stimuli and ensure they were grounded in theory and constructed to yield the information we desired.

Our results also extend fragmented recommendations from previous publications [30,32,33] and provide systematic qualitative and quantitative guidance to design an effective app-based intervention. We expanded previous recommendations for privacy and information security by providing detailed suggestions concerning handling of sensitive information.

### Limitations

This study has several limitations. First, focus groups were conducted using standard procedures and moderated by skilled individuals, but like all focus groups, the conversation was susceptible to bias and to swaying of individual opinions by dominant participants [40]. Second, the app framework was generated under the context of Chinese culture and the primary factors for child injury in China. The framework might need tailoring and adjustment if it were applied to other age groups, other diseases, or other countries.

### Conclusions

A theory-driven and evidence-based framework was established to guide the design of an app-based unintentional injury intervention program for the caregivers of Chinese children aged 0 to 6 years.

## Appendix

Multimedia Appendix 1 Procedure and discussion guide for focus groups.

Multimedia Appendix 2 Web-based survey questionnaire.

Multimedia Appendix 3 Data reporting guidelines, checklist for reporting results of internet E-Surveys (CHERRIES).

## Abbreviations

CHERRIES: checklist for reporting results of internet E-survey
COREQ: consolidated criteria for reporting qualitative research
FRAME: Framework for the Rational Analysis of Mobile Education
mHealth: mobile health
TPB: Theory of Planned Behavior
