# Supplementary material for: Needs Analysis for a Parenting App to Prevent Unintentional Injury in Newborn Babies and Toddlers: Focus Group and Survey Study Among Chinese Caregivers
Source: JMIR Mhealth Uhealth. 2019 Apr 30;7(4):e11957. doi: 10.2196/11957 (PMC6658302; doi:10.2196/11957)
Supplement: Multimedia Appendix 1 [file mhealth_v7i4e11957_app1.docx]

# Multimedia Appendix 1. Procedure and discussion guide for focus groups

Operation procedure of the focus group

1. Introduce the project and the operating procedure;

2. Sign informed consent documents and complete the “General questionnaire”, including socio-demographic characteristics for both caregivers and their children and unintentional injury history for their children.

3. Start audio-tape after obtaining permission of participants;

4. Share stories and experiences on unintentional injury prevention among children ages 0-6 years;

5. Implement group discussion according to the discussion guide.

Discussion guide

1. Which kinds of unintentional child injury do you most worry about when you take care of children? Do you want to learn knowledge and skills on how to prevent common unintentional child injuries?

2. Have you ever learned about how to prevent unintentional child injuries? If yes, how did you access the knowledge? And how do you like the way in which you accessed the knowledge?

3. What is your opinion toward application (app)-based unintentional injury prevention?

4. What kinds of unintentional injury prevention knowledge do you want to use app-based intervention to learn?

5. When and how often do you prefer to receive app-based knowledge disseminations on unintentional child injury prevention? What do you think is the best duration for each dissemination, on average?

6. What type of interface do you expect for the app-based intervention?

7. Do you expect to consult experts (including interacting with them) during the intervention time period?

8. Do you agree to participate in a survey related to child safety? If yes, which way do you prefer (self-administered printed questionnaire vs. online survey)? How often do you expect to complete the survey? And how long do you expect it will take you to complete the survey?

9. What are your biggest concerns for using an app?
