# Supplementary material for: Needs Analysis for a Parenting App to Prevent Unintentional Injury in Newborn Babies and Toddlers: Focus Group and Survey Study Among Chinese Caregivers
Source: JMIR Mhealth Uhealth. 2019 Apr 30;7(4):e11957. doi: 10.2196/11957 (PMC6658302; doi:10.2196/11957)
Supplement: Multimedia Appendix 2 [file mhealth_v7i4e11957_app2.doc]

# Multimedia Appendix 2. Online survey questionnaire

1. Adult caregiver’s gender:

A. Male

B. Female

2. Adult caregiver’s age group:

A. ≤19 years

B. 20-29 years

C. 30-39 years

D. 40-49 years

E. ≥50 years

3. Child’s sex:

A. Male

B. Female

Note: If an adult caregiver takes care of more than one young child, only record information concerning the youngest child.

4. Child’s age group:

A. ≤1 year

B. 2-3 years

C. 4-6 years

5. Has your child experienced the following events in the past 3 months [mark all that apply]?

A. Contact with heat and hot substances causing injury

B. Exposure to inanimate mechanical forces causing injury

C. Falls causing injury

D. Transport accidents

E. Exposure to animate mechanical forces causing injury

F. Accidental threats to breathing [suffocation risk]

G. Exposure to smoke, fire and flames

H. Unintentional poisoning by or exposure to noxious substances

I. Unintentional drowning risk or submersion

J. Exposure to electrical currents

K. Other______________

L. All of the above (options A to J)

M. None of the above

6. How do you currently learn about child safety [mark all that apply]?

A. Parenting software

B. WeChat

C. Newspapers and magazines

D. News advertisements

E. Training lectures

F. Brochures

G. Communication with other parents

H. Listening to the radio or watching TV shows

I. Other ______________

J. No suitable way to learn.

7. How satisfied are you with the current learning channels about child safety you have available?

A. Very unsatisfied

B. Unsatisfied

C. Neither unsatisfied nor satisfied

D. Satisfied

E. Very satisfied

8. Which topics of child safety and injury prevention have you ever learned preventive strategies for in the past [mark all that apply]?

A. Contact with heat and hot substances, or exposure to smoke, fire and flames

B. Exposure to inanimate mechanical forces

C. Falls

D. Transport accidents

E. Exposure to animate mechanical forces

F. Accidental threats to breathing [suffocation risk]

G. Exposure to smoke, fire and flames

H. Unintentional poisoning by and exposure to noxious substances

I. Unintentional drowning and submersion

J. Exposure to electrical currents

K. Other______________

L. All of the above (options A to J)

M. None of the above

9. Do you have hopes to learn more about how to prevent child injuries?

A. No, I have no desire to learn more

B. Not really, I do not have a lot of desire to learn more

C. I am mixed on whether I want to learn more or not learn more

D. Yes, I have some desire to learn more

E. Yes, I definitely have desire to learn more

10. Which aspects of preventive knowledge would you liked to learn [mark all that apply]?

A. Contact with heat and hot substances

B. Exposure to inanimate mechanical forces

C. Falls

D. Transport accidents

E. Exposure to animate mechanical forces

F. Accidental threats to breathing [suffocation risk]

G. Exposure to smoke, fire and flames

H. Unintentional poisoning by and exposure to noxious substances

I. Unintentional drowning and submersion

J. Exposure to electrical currents

K. Other______________

L. All of the above

M. None of the above

11. Which forms of training would be best for you to learn preventive knowledge [mark all that apply]?

A. Plain text

B. Pictures

C. Short written statements with pictures

D. Video testimonials

E. Cartoon vignettes

F. Interactive games

G. Other______________

12. How often do you want to learn preventive knowledge?

A. Once a day

B. Twice a week

C. Once a week

D. Once a month

13. At what time of day do you want to learn preventive knowledge?

A. Morning

B. Noon

C. Afternoon

D. Night

E. Any time of day is fine

14. For each time you learn preventive knowledge, how much time would you want to spend in training?

A. ≤2 minutes

B. 3-5 minutes

C. 6-10 minutes

D. ≥11 minutes
