# Supplementary material for: Needs Analysis for a Parenting App to Prevent Unintentional Injury in Newborn Babies and Toddlers: Focus Group and Survey Study Among Chinese Caregivers
Source: JMIR Mhealth Uhealth. 2019 Apr 30;7(4):e11957. doi: 10.2196/11957 (PMC6658302; doi:10.2196/11957)
Supplement: Multimedia Appendix 3 [file mhealth_v7i4e11957_app3.docx]

# Multimedia Appendix 3. Data reporting guidelines, checklist for reporting results of internet E-Surveys (CHERRIES)

| ***Item category*** | ***Checklist item*** | ***This study*** |
| --- | --- | --- |
| **Design** | Describe survey design | Purposive sample. Participants were recruited from users of the "La Ma Xue Yuan" WeChat account group, which is targeted primarily toward caregivers of children ages 0-6 years old. There are 9,290 users in the group, and they live in 35 provinces/autonomous regions/municipalities in China. We obtained further research subjects through a snowball sampling method, encouraging initial participants to forward and share information about the study until the number of caregivers participating in the survey plateaued, at which point the investigation was halted. |
| **IRB approval and informed consent process** | IRB approval | This study was approved by the Medical Ethics Committee of Institute of Clinical Pharmacology, Central South University [registration number CTXY-150002-4, February 27th, 2015]. |
|  | Informed consent | Informed consent was provided by completing the e-survey. |
|  | Data protection | This study does not involve personal or family privacy. The research data were collected, cleaned and analyzed anonymously. The data will be used only for scientific research and will be kept strictly confidential. |
| **Development and pre-testing** | Development and testing | Based on the results of the focus groups and internal discussion among the research group, the electronic survey plan was determined. It was grounded in theory. Further, before the formal large-scale investigation, 20 caregivers of preschool children were recruited to complete the questionnaire and offer their opinions about the items, permitting modification and improvement based on an early pilot test. |
| **Recruitment process and description of the sample having access to the questionnaire** | Open survey versus closed survey | Closed survey on the "La Ma Xue Yuan" official account. |
|  | Contact mode | Contact with participants online through WeChat. |
|  | Advertising the survey | Advertised initially on the “La Ma Xue Yuan” WeChat account group, and then forwarded and shared using snowball techniques via WeChat. |
| **Survey administration** | Web/E-mail | Online questionnaires online via WeChat. |
|  | Context | All users who are caregivers of children ages 0-6 were invited to participate. They completed the survey in their preferred location and at their preferred time. |
|  | Mandatory/voluntary | Voluntary survey. |
|  | Incentives | After completing the questionnaire, participants were entered into a “lucky draw”. In this survey, 150 lucky red envelopes were available, each totaling 200 Chinese yuan in cash. |
|  | Time/date | November 2016. |
|  | Randomization of items or questionnaires | No randomization. |
|  | Adaptive questioning | N.A. |
|  | Number of items | 14 items in total. |
|  | Number of screens (pages) | 1 Webpage per survey. |
|  | Completeness check | Completeness checks were conducted electronically, through www.wjc.cn |
|  | Review step | Respondents were able to review and change their answers if they wished. |
| **Response rates** | Unique site visitor | Unique site visitors were determined by user ID and repeat visitors were excluded. |
|  | View rate (ratio of unique survey visitors/unique site visitors) | Not available. |
|  | Participation rate (ratio of  unique visitors who agreed to participate/unique first  survey page visitors) | Not available. |
|  | Completion rate (ratio of users who finished the survey/users who agreed to participate) | In total, 1505 valid questionnaires were collected through the online survey. Due to privacy concerns, information about users who consented or started the survey but did not finish is unavailable. |
| **Preventing multiple entries from the same individual** | Cookies used | No cookies were used. |
|  | IP check | Duplicate database entries having the same user ID were eliminated before analysis. |
|  | Log file analysis | Duplicate database entries having the same user ID were eliminated before analysis. |
|  | Log file analysis | Duplicate database entries having the same user ID were eliminated before analysis. |
|  | Registration | N.A. |
| **Analysis** | Handling of incomplete questionnaires | Only completed questionnaires were analyzed. |
|  | Questionnaires submitted with an atypical timestamp | No questionnaires were submitted with atypical timestamps. |
|  | Statistical correction | If values of any of the independent variables included missing data, the entire case was excluded for the analysis |
